# Effects of Short-Term Concurrent Training Cessation on the Energy Cost of Running and Neuromuscular Performances in Middle-Distance Runners

**DOI:** 10.3390/sports9010001

**Published:** 2020-12-22

**Authors:** Nicolas Berryman, Iñigo Mujika, Laurent Bosquet

**Affiliations:** 1 Département des Sciences de l’activité Physique, Université du Québec à Montréal, 141 Avenue du Président Kennedy, Montréal, QC H2X 1Y4, Canada; 2 Institut National du Sport du Québec, 4141 Avenue Pierre-de-Coubertin, Montréal, QC H1V 3N7, Canada; 3 Lab. MOVE (EA6314), Faculty of Sport Sciences, University of Poitiers, 86000 Poitiers, France; laurent.bosquet@univ-poitiers.fr; 4 Department of Physiology, Faculty of Medicine and Nursing, University of the Basque Country, 48940 Leioa, Basque Country, Spain; inigo.mujika@inigomujika.com; 5 Exercise Science Laboratory, School of Kinesiology, Faculty of Medicine, Universidad Finis Terrae, Santiago H97R+8J, Chile

**Keywords:** strength training, running economy, detraining

## Abstract

Evidence supports the implementation of concurrent strength and running training, within the same mesocycle, to improve performances in middle- and long-distance events. However, very little is known about the effects of concurrent training cessation. The purpose of this investigation was to describe the effects of 4 weeks of explosive strength training cessation after an 8-week concurrent training protocol. Eight runners completed this study, which first included either plyometric (*n* = 4) or dynamic weight training (*n* = 4) in addition to the usual running regimen. Explosive strength training was thereafter interrupted for 4 weeks, during which running sessions were maintained. Participants were tested at baseline, after concurrent training and after concurrent training cessation. The results suggest that the energy cost of running improvements observed after the intervention (−5.75%; 95% CI = −8.47 to −3.03) were maintained once explosive strength training was interrupted (−6.31%; 95% CI = −10.30 to −2.32). The results also suggest that neuromuscular performances were maintained after 4 weeks of concurrent training cessation, especially when tests were specific to the training intervention. Furthermore, a 3000m time trial revealed a similar pattern, with improvements after the concurrent mesocycle (−2.40%; 95% CI = −4.65 to −0.16) and after concurrent training cessation (−4.43%; 95% CI = −6.83 to −2.03). Overall, only trivial changes were observed for aerobic endurance and $\dot{V}O_{2}peak$. Together, these results suggest that short-term explosive strength training cessation might be beneficial and could be considered as a taper strategy for middle-distance runners. However, coaches and athletes must interpret these results cautiously considering the study’s low sample size and the very limited available literature in this domain.

## 1. Introduction

Detraining, defined as a partial or complete loss of training-related adaptations as a consequence of training load reduction or training cessation [1], represents a crucial factor to consider both from a sports performance perspective but also for the athletes’ overall health and well-being [2]. The effects of short-term training cessation (4 weeks or less) are associated with declines in numerous fitness outcomes. For instance, 3 to 4 weeks of strength training cessation could lead to significant reductions in force endurance, maximal power and maximal force [3]. In addition to neuromuscular losses, training cessation could lead to declines in cardiovascular adaptations [1]. Twelve days of training interruption led to $\dot{V}O_{2}max$ declines of more than 5% in well-trained cyclists [4], and aerobic endurance could be similarly affected [5]. Intriguingly, the energy cost of running (Cr) does not seem to be modified after short-term (2 weeks) training cessation in distance runners [6]. Cr, a crucial performance determinant in middle- and long-distance events, could be improved after strength training interventions concurrently implemented with a running program [7], provided that training variables are manipulated appropriately [8]. While a recent review reported that 2 to 4 weeks of concurrent training interruption leads to reductions in 1RM (7–10%), $\dot{V}O_{2}max$ (5–15%), vertical jump (3–5%) as well as agility and repeated sprint ability (1–5%) [9], much less is known about the effects of concurrent training cessation on Cr. Thus far, one study including six elite male runners showed that the improvements in Cr attained after 12 weeks of concurrent training were maintained after 5 weeks of resistance training cessation [10]. 

In order to add information to this very limited but nevertheless important area of research, the purpose of this case report was to describe the effects of 4 weeks of explosive strength training cessation after an 8-week concurrent training protocol. We expected that the training-related benefits on Cr would be maintained after training cessation despite reductions in lower body maximal power.

## 2. Materials and Methods

### 2.1. Experimental Approach to the Problem

This investigation is a follow-up of an intervention study published by our research team, investigating the effects of explosive strength training on the energy cost of running [11]. In the original version of the study, concurrent training was followed by 4 weeks of strength training cessation, during which running sessions were maintained. Due to a major dropout rate (more than 40%) after the 1st cohort, the training cessation part was cancelled. Nonetheless, some participants from this 1st cohort completed the entire study, and their results are now presented in this case report. Briefly, participants were divided into 3 groups (plyometric training—PT, dynamic weight training—DWT or a control intervention—running only) for an 8-week concurrent training protocol, which was immediately followed by 4 weeks of explosive strength training cessation. Participants were therefore tested at baseline, after the concurrent training protocol and after the training cessation period. This study was reviewed and approved by the Research Ethics Board at Université de Montréal, where the study was conducted. All subjects were informed about the risks and benefits of the investigation and then provided informed consent before participating in the study.

### 2.2. Subjects

Eight participants (*n* = 4 for each DWT and PT) completed the entire study, including the concurrent training cessation part. However, no participants from the control intervention completed the training interruption period. Participants were well-trained male runners (see Table 1 for baseline performance characteristics) but had no experience in strength training. All participants had experience in different amateur running competitions.

### 2.3. Testing Protocol

All tests were performed at the same hour of the day, and organized into three testing sessions separated by at least 48 h, using the following sequence: (1) Cr and $\dot{V}O_{2}peak$, (2) lower-body maximal power, (3) countermovement jumps (CMJ) and the 3000 m time trial. Testing at all 3 time points (pre, post and follow-up) included measurements of key aerobic indices of running performance [12] and a 3000 m time-trial. $\dot{V}O_{2}peak$ (mL·kg^−1^·min^−1^) was measured (Moxus, AEI Technologies, Naperville, IL, USA) during an incremental test until exhaustion on a treadmill (Quinton, VA, USA), which also led to the identification of peak treadmill speed (PTS). Aerobic endurance represented the ratio (%) between the average speed maintained during the 3000 m time-trial and PTS [13]. The 3000 m time-trial was performed on a 200 m indoor track. Furthermore, lower body neuromuscular performances were assessed. A force-velocity squat test was completed on a guided rack and maximal power (W) was measured using a linear encoder (MuscleLab, Ergotest, Langesund, Norway). Vertical jump height (cm) was measured with an optical system (Optojump, Microgate, Bolzano, Italy) during a CMJ. Body mass index (kg/m^2^) was also measured at each time point.

### 2.4. Training Protocol

During the concurrent training period, PT and DWT participants included 1 explosive strength training session weekly in addition to their normal running regimen. Strength training load was equivalent for both experimental groups with 3 to 6 sets of 8 repetitions completed during each session. While PT participants executed drop jumps with a starting box height that optimized vertical jump performance, the DWT protocol consisted of concentric-only jumps with a load that optimized power output. Feedback was provided after each repetition to control intensity as the objective was to achieve 95% of peak performance for each repetition (peak power established during a force-velocity test for DWT; maximal vertical jump height for PT). The running program, equivalent between experimental groups [11], included 3 weekly sessions, with an emphasis on (1) maximal aerobic speed, (2) intermittent aerobic endurance and (3) continuous aerobic endurance. All training sessions were separated by at least 24 h.

### 2.5. Statistical Analyses

Considering the small sample size, a descriptive approach was prioritized. First, all individual scores were reported. Effect sizes (Hedges’ g) were calculated as described previously [14] using absolute scores for the entire sample (*n* = 8) but also for each training intervention. Interpretation was based on Cohen’s scale [15] where the effect was considered trivial (g < 0.20), small (0.20 ≤ g < 0.50), moderate (0.50 ≤ g < 0.79), or large (g ≥ 0.80). Moreover, individual relative changes (RC) from pre- to post concurrent training and from pre to follow-up were calculated ((post-pre)/pre * 100). Confidence intervals (95% CI) were then computed based on these relative changes. Cr changes were interpreted considering the smallest detectable difference (≅2%) as well as the typical adaptations observed after concurrent training interventions (2–8%) [7].

## 3. Results

**Figure 1:**
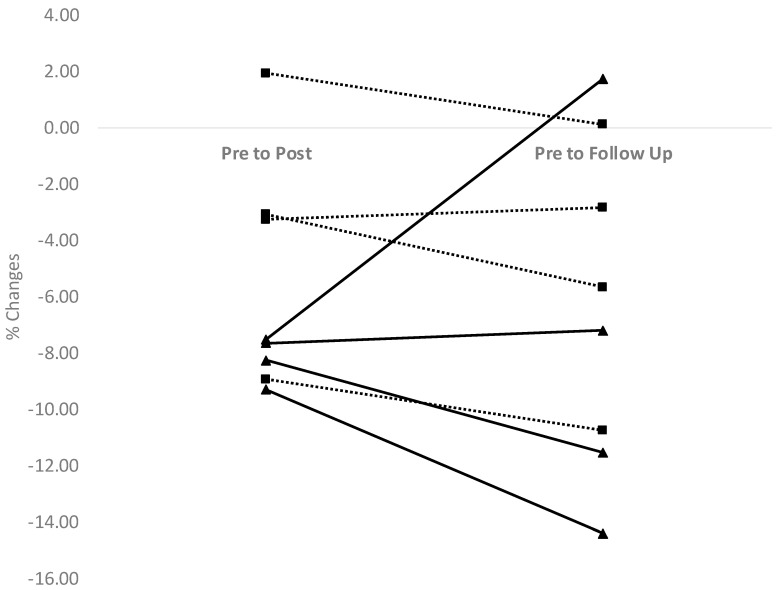
Individual % changes in energy cost of running. Plyometric training: solid lines. Dynamic weight training: dashed lines.

Analyses for the entire sample (*n* = 8) revealed that Cr was moderately improved from pre to post concurrent training (g = −0.57; RC (95% CI) = −5.75 (−8.47 to −3.03)) and these changes were maintained after the 4-week cessation period as suggested by a moderate pre to follow-up effect size (g = −0.61; RC (95% CI) = −6.31% (−10.30 to −2.32)). When focusing on the training modality (Table 1), PT resulted in large Cr reductions (g = −0.95; RC (95% CI) = −8.18% (−8.97 to −7.38)) whereas effects were considered small for DWT (g = −0.38; RC (95% CI) = −3.33% (−7.68 to 1.02)). From pre to follow-up, reductions were considered small for PT (g = −0.42; RC (95% CI) = −7.84% (−15.82 to 0.13)) and moderate for DWT (g = −0.55; RC (95% CI) = −4.78% (−10.01 to 0.45)). Figure 1 shows that seven runners improved Cr by at least 2% after the 8-week concurrent training intervention. From pre to follow-up, six runners had scores that were at least 2% lower (indication of improvements) than during baseline testing.

Small improvements in maximal power assessed during the force-velocity test were found after the 8-week concurrent training protocol (g = 0.33; RC (95% CI) = 7.98% (−3.10 to 19.07)). These changes were maintained after training cessation, as indicated by a small pre to follow-up effect size (g = 0.20; RC (95% CI) = 4.95% (−5.96 to 15.86)). However, specific training effects were observed. Indeed, as shown in Table 2, DWT led to moderate improvements both from pre to post (g = 0.61; RC (95% CI) = 17.21% (9.23 to 25.19)) and from pre to follow-up (g = 0.57; RC (95% CI) = 16.36% (6.91 to 25.81)). For PT, trivial changes were observed from pre to post (g = −0.09; RC (95% CI) = −1.25% (−18.33 to 15.83)), while small declines were found from pre to follow-up (g = −0.29; RC (95% CI) = −6.46% (−17.98 to 5.06)).

Similarly, small improvements in CMJ performance were found from pre to post concurrent training (g = 0.46; RC (95% CI) = 9.12% (5.46 to 12.78)) and from pre to follow-up (g = 0.28; RC (95% CI) = 7.80% (4.42 to 11.17)). When analyses were made based on the training intervention, DWT led to small and trivial changes from pre to post (g = 0.27; RC (95% CI) = 8.71% (3.33 to 14.10)) and from pre to follow-up (g = 0.08; RC (95% CI) = 6.53% (1.44 to 11.62)), respectively. Moreover, PT resulted in small improvements for both pre to post (g = 0.37; RC (95% CI) = 9.43% (3.77 to 15.10)) and pre to follow-up (g = 0.30; RC (95% CI) = 8.75% (3.84 to 13.66)).

**Figure 2:**
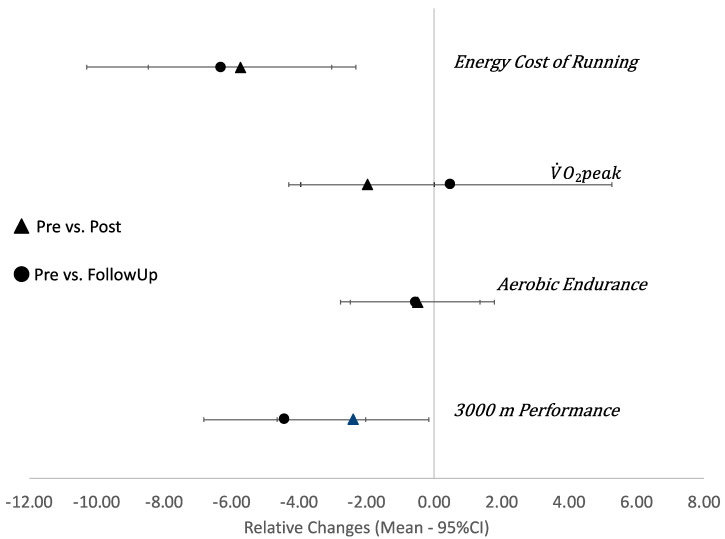
Relative changes for running performances. Energy cost of running and $\dot{V}O_{2}peak$: *n* = 8. Aerobic endurance and 3000 m performance: *n* = 7.

Overall (*n* = 8), only trivial changes (g < 0.20) were observed for other performance variables ($\dot{V}O_{2}peak$, aerobic endurance) in both pre to post and pre to follow-up comparisons. PT participants showed small reductions in $\dot{V}O_{2}peak$ from pre to post (g = −0.31) as well as from pre to follow-up (g = −0.20). However, small improvements were observed for the 3000 m performance test (see Figure 2) after concurrent training (g = −0.24; RC [95% CI] = −2.40% [−4.65 to −0.16]) and also after strength training cessation (i.e., pre to follow-up; g = −0.48; RC [95% CI] = −4.43% [−6.83 to −2.03]).

## 4. Discussion

The key outcome of this investigation was the observation of a 6.31% improvement (95% CI: −10.30 to −2.32) in Cr from baseline testing to concurrent training cessation. Considering it was improved by 5.75% (95% CI: −8.47 to −3.03) after the concurrent training intervention (pre to post), Cr was maintained after 4 weeks of concurrent training cessation. These changes fall within the typical range of Cr improvements observed in concurrent training studies (2–8%). Moreover, seven and six runners had improvements greater than 2% from pre to post and from pre to follow-up, respectively, suggesting that these changes were real and could therefore be utilized by sports scientists and coaches. From an individual perspective, only one runner (D2, Table 1) had negative adaptations from pre to post, and another participant (P4, Table 1) showed an important deterioration in Cr after the training cessation protocol. Such an observation once again shows the importance of an individualized approach to the assessment of the effects of concurrent training and its cessation.

In line with previous reports, the aerobic endurance parameters reported in this study as well as $\dot{V}O_{2}peak$ were not modified, neither after concurrent training nor after its cessation when the entire sample was analyzed [7,16]. However, some participants showed declines in these aerobic indices, which might be an occasion to further highlight the importance of individualization when it comes to concurrent training prescription.

Contrary to previous reports [3], the results found in this sample of runners suggest that neuromuscular performances were maintained after 4 weeks of concurrent training cessation, especially when tests were specific to the training intervention (CMJ for PT and maximal power for DWT). Although explosive strength training was removed from the training program, all participants maintained their running activities and it might have been a sufficient stimulus to avoid the negative effects of full training cessation, at least for 4 weeks.

Finally, the observation of a tendency towards greater 3000 m time trial improvements from baseline to follow up compared to pre vs. post concurrent training suggest that, overall, runners in this study did benefit from the 4-week concurrent training cessation. In this report, five participants out of seven who completed the follow-up time trial ran faster than during the post training time point. Intriguingly, this observation points towards a potential periodization strategy in which concurrent training cessation could be planned in order to optimize the athletes’ pre-competitive taper. However, the observation that performance in a 5 km time trial improved after 6 weeks of concurrent training before going back to baseline values after 6 weeks without strength training [17] suggests that concurrent training duration and subsequent interruption length are key factors for an optimal periodization strategy.

Aside from the small sample size, a limitation of this report is related to the absence of participants from a control intervention (only running sessions throughout the entire protocol). Until further reports are published, coaches and athletes are therefore invited to consider cautiously the available literature on that topic. Nevertheless, the actual report adds to a quite limited but still crucial area of research.

## 5. Conclusions

This brief report suggests that Cr improvements observed after an 8-week concurrent training period were maintained despite a short-term (4-weeks) explosive strength training interruption. Notably, these concurrent training cessation effects were obtained with a previously low-volume/high-intensity strength training protocol in moderately to well-trained male middle-distance runners. Short-term concurrent training cessation could be considered by coaches and athletes as a potential tapering strategy, but further research is required.

## Figures and Tables

**Table 1:** Aerobic indices of running performances.

	Cr (kcal·kg^−1^·km^−1^)			$\dot{V}O_{2}peak$ (mL·kg^−1^·min^−1^)			AerEnd (%)			3000 m(s)		
	Pre	Post	FU	Pre	Post	FU	Pre	Post	FU	Pre	Post	FU
P1	1.21	1.11	1.07	63.4	58.9	57.7	85.6	90.8	85.4	765	700	703
P2	1.15	1.06	1.07	66.9	64.9	66.0	88.6	87.3	91.9	677	669	653
P3	1.07	0.97	0.91	64.0	64.0	61.2	92.6	92.4	93.1	648	632	627
P4	1.21	1.12	1.23	56.6	55.6	61.7	88.1	86.0	85.7	791	785	764
Mean	1.16	1.06	1.07	62.7	60.9	61.7	88.7	89.1	89.0	720.3	696.5	686.8
SD	0.07	0.07	0.13	4.4	4.4	3.4	2.9	3.0	4.1	68.6	65.2	60.4
ES		−0.95 ^a^	0.03 ^b^		−0.31 ^a^	0.15 ^b^		0.09 ^a^	−0.02 ^b^		−0.26 ^a^	−0.10 ^b^
			−0.42 ^c^			−0.20 ^c^			0.05 ^c^			−0.34 ^c^
D1	1.00	0.97	0.97	50.7	51.4	55.3	89.3	86.8	85.4	780	778	790
D2	1.02	1.04	1.02	54.1	51.9	57.3	83.3	83.1	83.8	810	812	758
D3	0.96	0.93	0.90	72.2	70.4	67.9	91.8	91.8	NA	574	560	NA
D4	1.13	1.03	1.01	56.6	57.3	56.9	90.6	88.6	89.4	769	739	711
Mean	1.03	0.99	0.98	58.4	57.8	59.4	87.8	86.2	86.2	786.3	776.3	753.0
SD	0.07	0.05	0.05	9.5	8.9	5.8	3.9	2.8	2.9	21.2	36.5	39.7
ES		−0.38 ^a^	−0.21 ^b^		−0.05 ^a^	0.09 ^b^		−0.16 ^a^	0.01 ^b^		−0.13 ^a^	−0.35 ^b^
			−0.55 ^c^			0.03 ^c^			−0.24 ^c^			−0.57 ^c^

Each line (P1–P4 and D1–D4) corresponds to a participant. Cr: Energy Cost of Running, AerEnd: Aerobic Endurance, FU: Follow-Up, ES ^a-b-c^: Effect size a—Post vs. Pre, b—FU vs. Post, c—FU vs. Pre, NA: Data non-available at FU, so participant (D3) excluded from Means, SD and ES calculations.

**Table 2:** Anthropometry and neuromuscular performances.

	Age (Years)	BMI (kg/m^2^)			Maximal Power (W)			CMJ (cm)		
		Pre	Post	FU	Pre	Post	FU	Pre	Post	FU
PT										
P1	22	24.0	24.2	23.7	1446.2	1296.2	1266.8	39.1	40.1	41.8
P2	23	20.0	20.5	20.2	985.7	1222.7	1095.6	36.7	41.0	38.4
P3	24	24.4	23.5	23.5	979.9	835.9	852.4	29.7	31.9	31.9
P4	39	26.3	25.5	25.5	1223.0	1174.5	1081.3	26.8	31.1	31.1
Mean	32.0	23.7	23.4	23.2	1158.7	1132.3	1074.0	33.1	36.0	35.8
SD	11.2	2.6	2.1	2.2	222.6	203.9	170.1	5.8	5.3	5.2
ES		−0.05 ^a^	−0.06 ^b^			−0.09 ^a^	−0.19 ^b^		0.37 ^a^	−0.03 ^b^
			−0.07 ^c^				−0.29 ^c^			0.30 ^c^
DWT										
D1	37	24.1	24.5	24.1	968.6	1201.8	1206.9	27.4	29.0	30.4
D2	27	27.7	25.7	25.0	1359.7	1446.9	1414.4	35.9	41.0	38.3
D3	35	21.2	21.2	21.2	1012.1	1168.8	1147.5	37.6	39.3	NA
D4	33	24.7	24.4	24.2	1373.0	1687.0	1694.7	41.0	43.5	41.8
Mean	33.0	24.4	23.9	23.6	1178.4	1376.1	1365.9	34.8	37.8	36.8
SD	4.3	2.7	1.9	1.7	217.9	241.5	247.3	6.9	7.8	5.8
ES		−0.11 ^a^	−0.07 ^b^			0.61 ^a^	−0.03 ^b^		0.27 ^a^	−0.04 ^b^
			−0.17 ^c^				0.57 ^c^			0.08 ^c^

Each line (P1–P4 and D1–D4) corresponds to a participant. BMI: Body Mass Index, CMJ: countermovement jump, FU: follow-up, ES^a-b-c^: effect size a—post vs. pre, b—FU vs. post, c—FU vs. pre, NA: data non-available at FU, so participant (D3) excluded from means, SD and ES calculations.

## Data Availability

Data is contained within the article.
